# Evaluation of Indoor Radon Activity Concentrations and Controls in Dwellings Surrounding the Gold Mine Tailings in Gauteng Province of South Africa

**DOI:** 10.3390/ijerph20217010

**Published:** 2023-11-02

**Authors:** Paballo M. Moshupya, Seeke C. Mohuba, Tamiru A. Abiye, Ian Korir

**Affiliations:** 1 School of Geosciences, University of the Witwatersrand, Johannesburg 2050, South Africa; seekecarol@gmail.com (S.C.M.); tamiru.abiye@wits.ac.za (T.A.A.); 2 Centre for Nuclear Safety and Security, National Nuclear Regulator, Centurion 0046, South Africa

**Keywords:** indoor radon (Rn-222), mine tailings, solid-state nuclear track detectors, South Africa

## Abstract

Radon in dwellings is recognized as the primary source of natural radiation exposure to members of the public. In the West Rand District and Soweto in the Gauteng Province (South Africa), indoor radon (^222^Rn) mapping was carried out to assess the exposure levels of radon in dwellings around gold and uranium mining tailings dams. This study was conducted predominately during warm and cold seasons, using the solid-state nuclear track detectors. In summer months, the indoor radon levels measured in all areas ranged from below the lower limit of detection to 71 Bq/m^3^, with a mean value of 29 Bq/m^3^, whereas in winter, the levels ranged between 11 and 124 Bq/m^3^, with a mean value of 46 Bq/m^3^. Higher indoor radon levels are found in colder months (winter season) than warmer months (summer season). However, no dwellings with indoor radon levels that exceed the WHO (2009) recommended reference level of 100 Bq/m^3^ were found, except for one that was constructed directly on soil mixed with tailings material. It is recommended that residents should keep their indoor radon levels low through continuous ventilation so as to minimize the buildup of radon and the likelihood of increased health hazards associated with radon exposure.

## 1. Introduction

Radon (^222^Rn) is naturally found in the environment in both outdoor and indoor settings; however, in indoor environments, radon yields higher exposures to members of the public than in outdoor environments [1]. This is mainly because radon disperses rapidly in outdoor air, whereas in enclosed spaces, it accumulates to higher concentration levels [2]. Exposure to indoor radon is identified as the primary source of natural radiation exposure to the public [3] and accounts for 50% of the annual radiation exposure received by humans [4]. 

Exposure to radon results in increased health risks to the human population. When radon decays, it forms ^218^Po and ^214^Po, which are respirable solid particles that become attached to aerosol and dust particles in the air [5]. When inhaled, these short-lived decay products irradiate the lung lining tissues with alpha particles and ultimately cause lung cancer [2,6]. At present, radon is considered the second largest cause of lung cancer [7]. The chances of developing lung cancer due to radon are also dependent upon other factors, such as the amount of dose received, the duration of exposure, smoking habits and exposure to other lung cancer-causing agents [6,8]. Individuals exposed to high radon concentrations are at a high risk of developing lung cancer; also, long term exposure to low concentrations results in increased lung cancer risks [9,10]. 

In indoor environments, the occurrence of radon results from a combination of different sources and depends upon multiple factors. Radon is derived primarily from the radioactive decay of uranium (^238^U). In a typical house, the primary potential sources that contribute to indoor radon are uranium- and radium-bearing soils and rocks, building materials, domestic water, natural gas and outdoor air [11,12]. The higher the uranium concentration in an area, the greater the probability of high radon accumulation in indoor air [13]. Studies have found that relatively high radon concentrations in indoor air are commonly associated with granites [14,15], shales [16,17], soils and permeable unconsolidated sediments rich in uranium and radium [18,19]. Human activities such as the mining and processing of economic minerals such as uranium, gold, phosphorous, oil and gas, mineral sands and coal combustion significantly increase the amount of radon released into the environment [20,21]. Once radon is released into indoor environments, it becomes trapped and accumulates in the building. The amount of radon gas that accumulates in a building depends on the volumetric dimensions of the building and the ventilation rate [22].

In South Africa, the historic mining and processing of gold and uranium led to the deposition of large tailings dams, which are enriched with long-lived, naturally occurring radioactive elements, and are considered to be potential sources of radon [23,24,25,26]. A study conducted in some parts of the West Rand region, particularly in the Krugersdorp area, revealed that the gold mine tailings result in outdoor radon levels ranging between 32 Bq/m^3^ and 1069 Bq/m^3^ [26]. Also, studies that assessed radioactivity levels in mine tailings dams in the West Rand region found that there are particular mine tailings with high activity concentrations of ^238^U, which is the parent radionuclide of ^222^Rn [27,28]. The concerning issue is that there is a greater number of dwellings that are in close proximity to the mine tailings dams in Gauteng Province. Under such circumstances, radon emanating from the mine tailings may be dispersed into the surrounding dwellings. Also, tailings material with a high concentration of uranium-238 and associated decay products may be eroded to nearby residential areas and result in elevated radon indoor concentrations in the nearby vicinity. Currently, there is still a lack of studies on indoor radon exposures in areas that are in close proximity to gold mine tailings. Hence, this study’s aim was to evaluate the indoor radon activity levels on a regional scale in the vicinity of the gold mining regions of Gauteng Province in South Africa, and to assesses the impact of mine tailings dams, as well as other parameters, on indoor radon concentrations. 

### 1.1. Study Area

**Figure 1:**
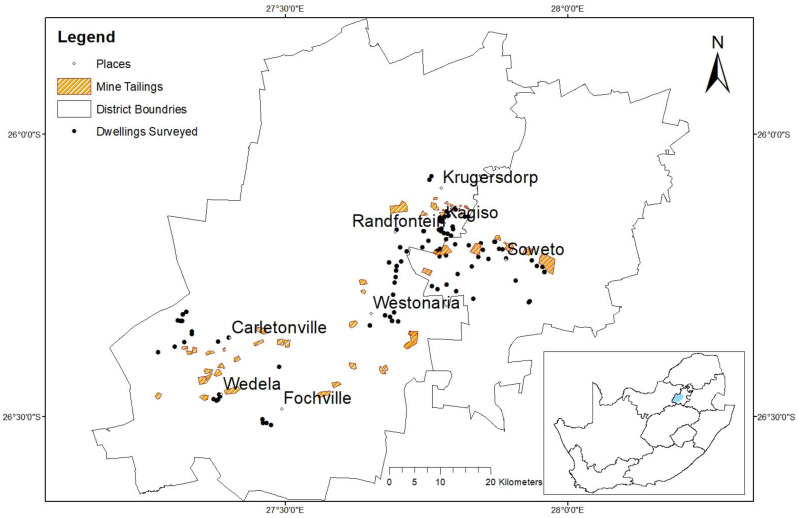
Map of the study area and locations surveyed for indoor radon.

The study area is located in the north–central part of South Africa in the Gauteng Province (Figure 1). The area forms part of the West Rand District Municipality and Soweto in the Johannesburg Metropolitan Municipality. These areas are dominated by mine residual products from gold and uranium mining activities. The main residential areas of focus for the indoor radon mapping survey fall in the major townships of Krugersdorp, Randfontein, Westonaria, Fochville, Wedela, Carletonville and Soweto that are located close to mine tailings (Figure 1). The area was historically used for mining gold in the Witwatersrand Supergroup rocks, and there are still continuing operations at present. Uranium was also mined as a by-product of gold. The area is used mainly as a residential area, and most of the residential households in the region are surrounded by mine tailings dams, as shown in Figure 1.

### 1.2. Climate of the Area

**Figure 2:**
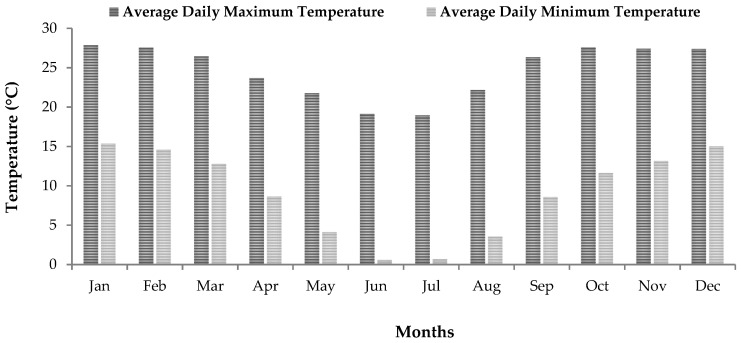
Mean maximum and minimum temperatures (data source: South African Meteorological Service: 2010–2023).

**Figure 3:**
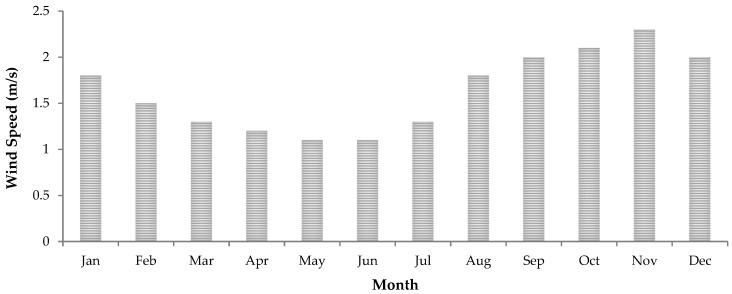
Average monthly wind speed (data source: South African Meteorological Service: 2010–2023).

The selected study area generally experiences warm and cold seasons, with an average maximum temperature of 28 °C and an average minimum temperature of 1 °C. The warm season is experienced between October and March, whereas the colder temperatures are experienced between May and August, with the coldest months being June and July (Figure 2). The area receives a mean annual rainfall of about 693 mm based on the recent rainfall data recorded from 2010 to 2023. The monthly average wind speed recorded throughout the day in the study area ranges between 1.1 and 2.3 m/s (Figure 3). 

### 1.3. Geology of the Area

**Figure 4:**
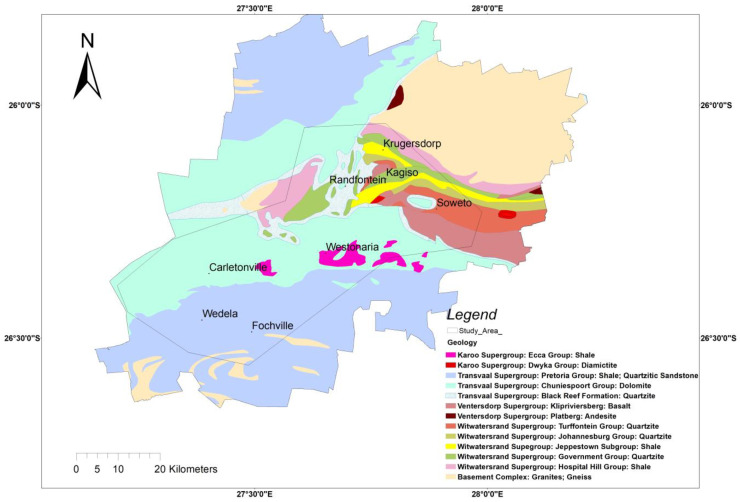
Geology of the study area and the extent of the area where indoor radon surveys were conducted.

On a regional scale, the geology comprises basement crystalline rocks, the metasedimentary rocks of the Witwatersrand Supergroup, the meta-volcanic rocks of the Ventersdorp Supergroup and the rocks of the Transvaal and Karoo Supergroups (Figure 4). In the extent of the selected study area where indoor radon measurements were conducted, the dominant rock types include the metasedimentary rocks of the Witwatersrand Supergroup that were deposited between 3074 and 2714 Ma [29]. The Witwatersrand Supergroup is divided into the West Rand Group and Central Rand Group. The West Rand Group rocks found in the extent of the studied area are the shales of the Hospital Hill and Jeppestown Subgroups and quartzite of the Government Group. The Central Rand Group rocks found in the area include the quartzites of the Johannesburg and Turffontein Subgroups. The area is also underlain by basalt of the Klipriviersberg Group and andesite of the Platberg Group that belong to the Ventersdorp Supergroup, which were deposited between 2714 and 2665 Ma [30]. The rocks of the Transvaal Supergroup are also dominant in the study area. These include the quartzites of the Black Reef Formation, Malmani dolomites of the Chuniespoort Group, as well as the shale and quartzitic sandstones of the Pretoria Group [31,32,33]. Succeeding the Transvaal Supergroup are the rocks of the Karoo Supergroup, which are exposed in the central eastern part of the study area (Figure 4). The rock types found in the study area that belong to the Karoo Supergroup are diamictites of the Dwyka Group and shale of the Ecca Group.

## 2. Materials and Methods

### 2.1. Instrumentation for Indoor Radon Measurements

There are various existing methods used for long-term measurements of indoor radon. The IAEA safety series on the design and conduct of indoor radon surveys defines long-term measurements as measurements conducted for a period of two months or longer [34]. The most common method applied for long-term indoor radon measurement is through the means of solid-state nuclear track detectors (SSNTDs), which typically consist of plastic polymers such as polyallyl diglycol carbonate (CR-39), cellulose nitrate (LR-115) or polycarbonate (Makrofol) [34]. The exposure of passive alpha track-etch detectors (SSNTDs) over months flattens out fluctuations in indoor radon concentrations caused by changes in meteorological conditions that occur within a short time [35]. SSNTDs are widely used in many countries that have conducted indoor radon mapping programs [7,34]. In this study, the solid-state nuclear track detectors (SSNTDs) were also used to the measure radon levels in dwellings. These detectors were supplied by Radosys, Ltd. (Budapest, Hungary), which develops and manufactures monitoring systems based on etched-track technology. They comprise a CR-39 chip, which is sensitive to alpha particles and operates in such a way that, when alpha particles from radon are generated, they leave microscopic tracks on the chip. Each detector is sealed into a radon-proof bag. The detectors have individual serial numbers for identification. The lowest level of detection (LLD) for the detectors used is about 4.5 kBqh/m^3^ (or 0.3 track/mm^2^). 

### 2.2. Indoor Radon Mapping Approach and Deployment Strategy

In the West Rand District and Soweto, the indoor radon measurements were conducted in areas dominated by gold and uranium mine tailings. The measurements were taken in close proximity to the mine tailings in dwellings that are situated less than 500 m from the tailings and extend to areas outside the mine tailings clusters. This strategy was employed to better assess the influence of mine tailings on indoor radon levels. The dwellings were selected randomly, and the measurements were conducted under normal living conditions and in higher occupancy areas in the household, such as bedrooms, living rooms and kitchens. Two (2) detectors were placed in randomly selected households to obtain the average representative concentration. However, in one-room dwellings, only one detector was placed. The detector was placed at a typical breathing height of 1 m to 1.5 m above the floor and about 0.5 m away from the wall. The detectors were placed approximately 1 m away from windows, doors and other potential openings to avoid interferences from outdoor air. The period of exposure for the detectors was 3 to 4 months to account for temporal variations and obtain average concentration levels. During the deployment of radon detectors, information related to the characteristics of the dwelling such as the building materials used for construction, ventilation, floor type and the age of the building structure were collected. 

The measurements were performed predominately during the warm and cold seasons to observe any seasonal variations. The first measurements were conducted from late October 2021 to January 2022 (warm season) and the second measurements were conducted from mid-June to early October 2022 (cold season). During the second survey, certain measurements were taken in houses that were previously measured during the warm season to observe seasonal variations under the same house characteristics and conditions. In total, 184 detectors were distributed in 100 dwellings during the first survey, which was performed during the warm months, and a total of 153 detectors from 85 locations were successfully retrieved and analysed. During the second survey that took place predominantly during the colder months, a total of 78 radon detectors were distributed in 39 dwellings, and a total of 65 detectors from 35 locations were successfully recovered and analysed. Figure 1 presents the locations where indoor radon measurements were conducted. Of all the measured dwellings, 23 were assessed during both the warm and cold seasons for seasonal variations. After the period of exposure lapses, the radon detectors were collected from the dwellings and submitted to Radosys, Ltd. Laboratory in Hungary for analysis. The main drawback associated with this study is the loss of some of the radon detectors that were placed within dwellings. This results in the recovery rate of the deployed detectors not being one hundred percent (100%).

### 2.3. Laboratory Analysis of Radon Detectors

The detectors were etched for 4 h and 30 min at 90 °C and thereafter were cleaned with an ultrasonic cleaner. Etching increases the visibility of the tracks on the sheet. After drying, the track densities on the sheets were determined using an RS Radometer V8. To determine the radon activity concentration, the following equations were used [36]:

Exposure (kBqh/m^3^) = CF × (TD_i_ − TD_0_) × F

And

Radon Activity Concentration (RAC)(Bq/m^3^) = Exposure × 1000/(24 × T)

where

CF is the batch-specific calibration factor (kBq/m^3^·h/(track/mm^2^));
TD_i_ is the chip’s track density;
TD_0_ the factory background;
T is the exposure time (days);
F is a multiplicative factor.

### 2.4. Data Analysis

Firstly, the values obtained in dwellings where two measurements were taken were averaged to obtain a representative mean radon concentration of the dwellings. Statistical analysis was performed to observe the general trend and distribution of the data for every area that was surveyed. The correlation of the indoor radon concentrations with various factors such as the distance of the dwellings from the mine tailings, underlying geology, concentrations of the parent radionuclides, seasonal variations and building characteristics was performed to better assess their relationship and influence on indoor radon levels.

The influence of the mine tailings on indoor radon was evaluated by determining variations in the radon concentrations with distance from the mine tailings zones. This was performed by estimating the distance of the measured dwellings from the closest mine tailings zone using the distance measure attribute in Google Earth that measures the distance between two points on the ground. Thereafter, linear regression was used to observe the correlation between the indoor radon levels and the distance from the mine tailings. 

Spatial analysis was also performed to better understand the distribution of indoor radon across the study area and correlate the indoor radon concentrations with the parent radionuclide, ^238^U. An indoor radon distribution map was constructed using the GIS inverse distance weighting (IDW) interpolation technique. This technique assumes that values that are close to each other are more related than those that are further away. The interpolated value of an unsampled point is a result of the weighted average of known neighboring values, and the weights are inversely associated with the distances between the sampled and predicted locations [37]. The IDW technique uses the following mathematical formula [38]:

$$Z=\frac{\sum_{i=1}^{n}\frac{1}{{\left(d_{i}\right)}^{p}}Z_{i}}{\sum_{i=1}^{n}\frac{1}{{\left(d_{i}\right)}^{p}}}$$

where

*Z* is the estimated value for the prediction point;
*Z_i_* is the measured value of the sampling point;
*d_i_* is the distance between the sample and prediction point;
p is a power parameter;
*n* is the number of sampling points.

The IDW interpolation technique yields better results when the sampling points are distributed regularly than when they are clustered [38]. Hence, this technique was used in this study, as the sampling points were well distributed.

## 3. Results

### 3.1. Indoor Radon Activity Concentration

**Figure 5:**
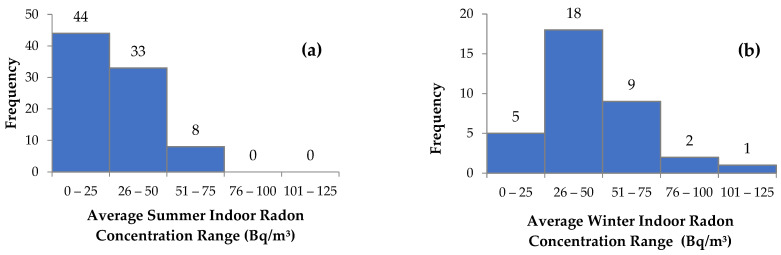
Average indoor radon activity concentration for the summer (**a**) and winter (**b**) measurements.

The results of the indoor radon measurements performed during the summer months in dwellings that are surrounded by the gold mine tailings in the West Rand District and Soweto are shown in Table 1. In the West Rand District, the average indoor ^222^Rn concentrations ranged from below the lower limit of detection (LLD) to 69 Bq/m^3^, with a mean value of 27 Bq/m^3^, whereas in Soweto, the levels ranged from 15 to 71 Bq/m^3^, with a mean value of 34 Bq/m^3^. The activity concentrations of most of the dwellings that were monitored in summer (52%) fell between a range of 0 and 25 Bq/m^3^, as shown in Figure 5a. During the winter months, the overall average activity concentration ranged from 11 to 124 Bq/m^3^ (Table 1). In most of the measured dwellings, the activity concentrations fell between 26 and 50 Bq/m^3^ in winter (Figure 5b). The data indicate that there are variabilities between the indoor radon concentrations in the winter and summer months. In winter, the mean value of the indoor radon concentrations was found to be 46 Bq/m^3^, whereas in summer, it was 29 Bq/m^3^. In addition, average radon concentrations above 75 Bq/m^3^ were recorded in winter, whereas in summer, the average activity concentration in all measured dwellings was below 75 Bq/m^3^ (Figure 5). 

### 3.2. Seasonal Variations on Indoor Radon

**Figure 6:**
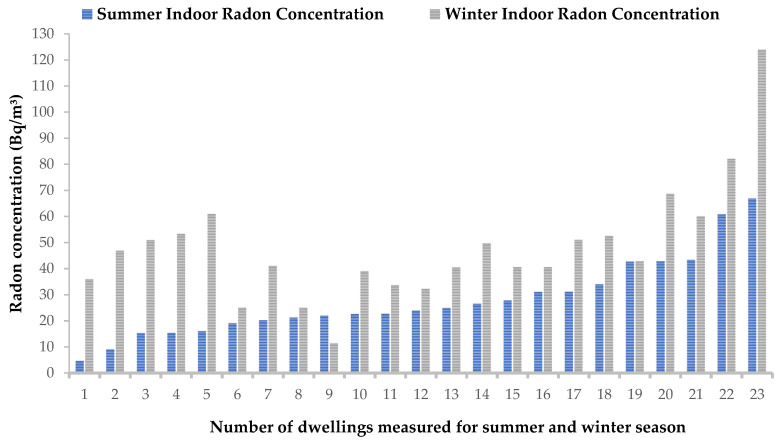
Comparison of summer and winter indoor radon results obtained from the dwellings measured during both seasons.

The meteorological conditions in the selected area of study vary significantly between the warm and cold seasons, as depicted in Figure 2. To determine the effects of seasonal variations on indoor radon, the measurements that were recorded in the same dwellings in both the warm and cold months were compared. This was to ensure that comparisons were made under the same geographical and environmental conditions, house characteristics and living habits. Furthermore, the comparisons were made to obtain the region-specific correction factor that could be used for seasonal adjustments of the radon measurements performed in summer. The data obtained indicate that there was a significant increase in indoor radon concentrations during the winter months (Figure 6). The average ratio of winter to summer indoor radon levels was determined for the 23 dwellings that were measured for seasonal variations, and it was found to be 1.7. This means that, on average, the indoor radon levels during winter are almost twice the summer activity concentrations, and therefore, could imply that the rate of ventilation which changes with weather conditions could be a contributing factor to the observed seasonal variations in indoor radon concentrations. In winter, the ventilation rate is low, as windows and other house openings are mostly kept closed, which therefore leads to high indoor radon accumulation due to low occurrences of indoor–outdoor air mixing conditions.

It can be observed that the indoor radon levels obtained in the studied areas are subject to seasonal variations. Higher radon concentrations were recorded during winter than in the summer season. This phenomenon was observed in studies conducted in various regions [39,40,41,42]. The high variability in indoor radon concentrations in winter and summer may also occur as a result of changes in meteorological conditions, which, among other factors, have a direct impact on indoor radon concentrations [43,44]. During winter, there is a significant difference in temperature between outdoor and indoor environments, with high temperatures indoors. This results in a greater air density difference, which induces depressurization at the base of dwellings and, therefore, allows for the ingress of air into dwellings [44]. On the other hand, the high indoor radon concentrations that were found during winter correspond with the lower wind speeds found during this season (Figure 3), whereas the lower indoor radon concentrations found in summer correspond to the higher wind speed conditions experienced during the summer months (Figure 3). High wind speeds increase ventilation in a building and flush radon from soil gas, thus reducing the amount of radon available for transport into surrounding dwellings [45]. Hence, higher wind speeds are associated with lower indoor radon concentrations. These findings show that variations in wind speed and temperature during various seasons have a concurrent effect on indoor radon concentrations.

Based on the results presented in Table 2, it is observed that the differences in radon concentrations that were found in different rooms of the same dwelling in winter are not as significant as those found in summer. On average, the ratios of the radon concentrations found in two rooms of the same dwelling were 1.2 in winter and 2.8 in summer. The low variance observed in winter is because the exchange rate of radon between indoor and outdoor environments is low, therefore resulting in internal airflow that allows for the interchange of air between rooms with limited interference with outdoor air. The high variability observed in summer between different rooms is due to the high indoor–outdoor air circulation that occurs as a result of increased ventilation through the opening of windows and doors for long periods. It is apparent that the radon activity concentration in every room is dependent on the volumetric dimensions of the room as well as the rate and size of ventilating openings. This therefore indicates that, independent of the sources that contribute to radon in dwellings, the ventilation rate has a great effect in controlling the amount of radon that accumulates in indoor environments. In addition, these findings confirm that the rate of ventilation, which, to some extent, depends on seasonal conditions, also contributes to the seasonal indoor radon variations observed between winter and summer. 

### 3.3. Comparison of Indoor Radon Levels with Reference Levels

**Figure 7:**
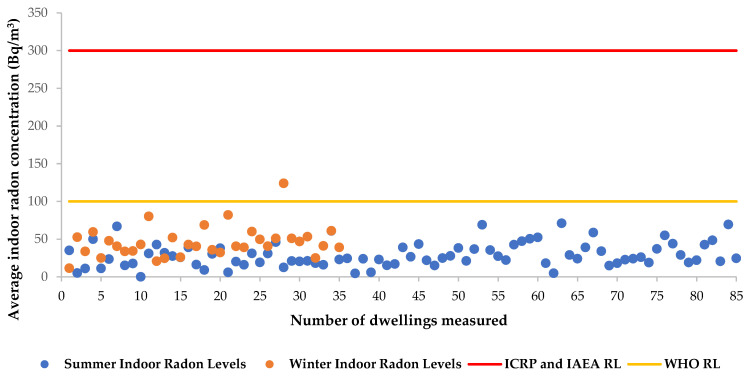
Comparison of summer and winter indoor radon activity concentrations with the recommended reference levels (RLs) for indoor radon exposures.

Various international agencies who are responsible for providing guidelines on radiological protection and policies to protect the health of the public have established reference levels for indoor radon exposures. The International Atomic Energy Agency (IAEA) and the International Commission on Radiological Protection (ICRP) recommend a reference level of 300 Bq/m^3^ for indoor radon exposure [46,47]. On the other hand, the World Health Organization (WHO) recommends a reference level of 100 Bq/m^3^ as a measure to reduce health hazards associated with indoor radon exposure, and further specifies that if this level cannot be achieved, the indoor radon value of 300 Bq/m^3^ should not be exceeded [7]. Based on the results of the indoor radon measurements that were performed in the summer months, it was observed that the average activity concentration obtained in all the measured dwellings were well below the recommended reference levels of 300 Bq/m^3^ and 100 Bq/m^3^ proposed by the ICRP, the IAEA and the WHO (Figure 7). Moreover, the winter indoor radon levels were also found to be well below the recommended reference level of 300 Bq/m^3^ [46,47]. Of all the dwellings surveyed in winter, only one dwelling had an average indoor radon concentration above the WHO reference value of 100 Bq/m^3^ (Figure 7). This value of 124 Bq/m^3^ was obtained in a dwelling located in Kagiso (Krugersdorp), where high uranium-238 (^238^U) concentrations were found in soil mixed with eroded materials from the nearby tailings [28]. Based on the information acquired during the study on the characteristics of the house, it was observed that the house has limited ventilation openings. 

### 3.4. Correlation of Indoor Radon with Distance from the Mine Tailings

**Figure 8:**
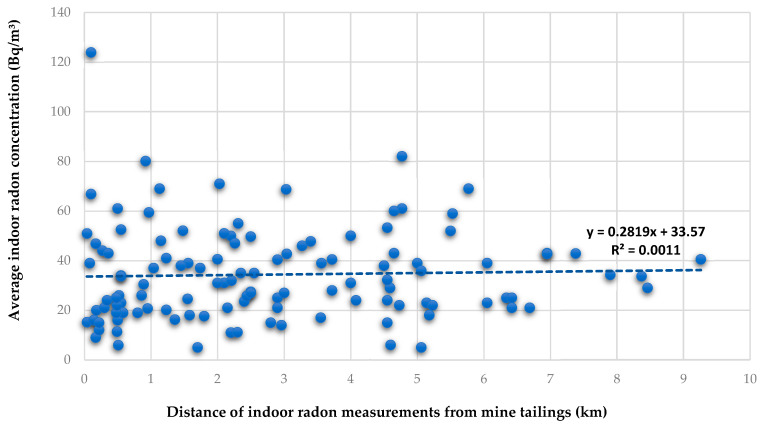
Distribution of indoor radon with distance from the mine tailings clusters.

Mining and processing economic minerals have the potential to contribute to radon releases in the environment. In a study conducted in some parts of the West Rand District, it was found that radon levels arising from the mine tailings ranged from 37.1 to 1068.8 Bq/m^3^ [26]. In addition, the studies that assessed the radioactivity levels in mine tailings in various parts of the West Rand District in Gauteng Province found that there are particular mine tailings with elevated concentrations of the radon parent radionuclide, ^238^U [27,28]. It was expected that the radon levels in dwellings that are located in close proximity to tailings may have high radon concentrations. The findings of this study, presented in Figure 8 and Table 3, show that there is no definite trend observed with indoor radon with the distance from the mine tailings. There is a wide range of variability in indoor radon concentrations for residential homes that are located within the same distance from the closest mine tailings (Figure 8). This observed pattern may be attributable to the high variability in radioactivity levels found in the tailings scattered throughout the study area. Unexpectedly, even dwellings that are located close to a mine tailing that releases high-activity concentrations of 1068.8 Bq/m^3^ do not have elevated or alarming indoor radon concentrations of above 300 Bq/m^3^ and may not require regulatory control. 

### 3.5. Indoor Radon Correlation with the Underlying Geology

**Figure 9:**
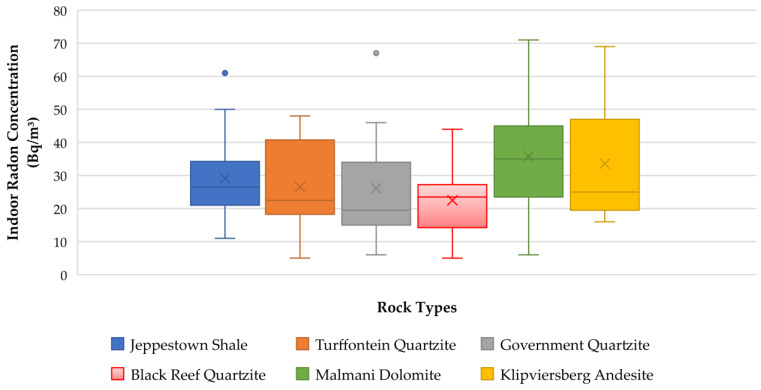
Correlation of indoor radon with the underlying geology.

Geology is the most significant contributing source of radon and its distribution in the environment [48]. The distribution of radon levels in dwellings constructed on various geological units was investigated in this study. The observed findings are presented in Figure 9. There are variations in indoor radon concentrations in each rock unit; however, the values in areas underlain by quartzites of various Subgroups are comparable. The variability observed between various rock types shows the effect of the geology on the distribution of radon. Although the overall indoor radon values are lower than the regulatory reference levels, dolomites, basalts and shales are characterized by higher indoor radon levels when compared to the quartzites of the Government Subgroup, Turffontein Subgroup and Black Reef Formation (Figure 9). The lowest indoor radon concentrations associated with quartzites are evidently a consequence of the low uranium-238 concentration found in this rock type, as presented in a study which characterised radioactivity levels in rocks [28]. In the same study [28], shales were found to have a higher content of ^238^U; hence, indoor radon levels were also found to be correspondingly higher. 

In areas underlain by carbonates such as dolomites and limestones, higher indoor radon concentrations are found due to high permeability conditions arising from karst environments, which then enhance the mobility of radon [49]. This phenomenon may also be the case in this study. The higher indoor radon levels in dolomitic areas may occur due to the possible dissolution of dolomites that may lead to subsurface voids and conduits that could serve as natural conduits for the migration of radon from the source rocks to the surface. The findings from the correlation with the underlying geology confirms that the type of bedrock found beneath the dwellings contributes to the variability in indoor radon concentrations. 

### 3.6. Radon Correlation with Building Characteristics (Ventilation and Age of the Buildings)

**Figure 10:**
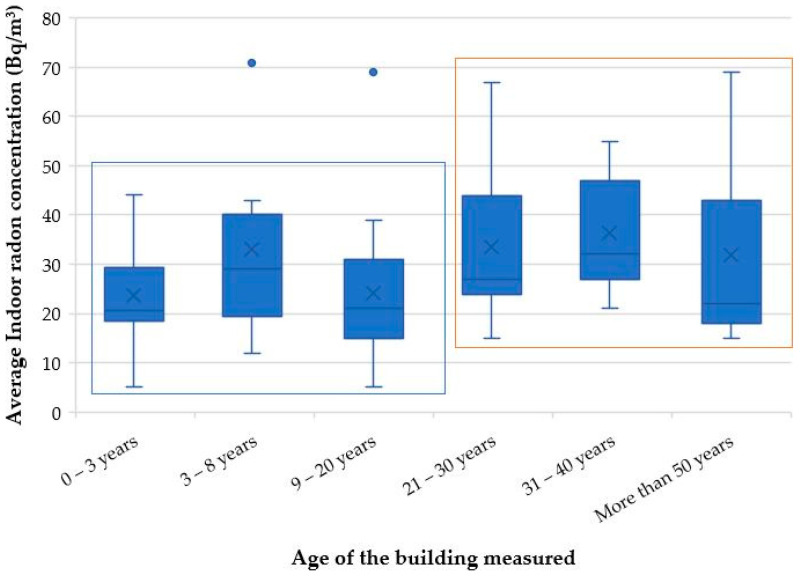
Correlation of indoor radon concentrations with the age of the measured dwellings.

The characteristics of a building structure play a significant role in the variability of radon concentrations found in indoor environments [49]. The entry rate and concentration level of indoor radon, among other factors, depend on the infiltration attributes of the building, the substructure air tightness and the architectural form of the dwelling [50]. Based on the site observations recorded during the indoor radon survey, most of the houses (81%) were characterized to have a good natural ventilation, with 19% of the dwellings having intermediate ventilation conditions. The generally low indoor radon concentrations found in this study (Figure 7) may be due to most of the dwellings measured being well ventilated. In Figure 10, the indoor radon concentrations are correlated with the age of the measured dwellings. It is found that indoor radon levels are slightly higher in older dwellings of greater than 20 years when compared to newer structures. This may be because older buildings are prone to ageing effects such as cracks, fractures and small openings in the floor slabs and walls that may increase entry points of radon gas from the soil beneath the house, therefore increasing the concentration levels of indoor radon.

### 3.7. Spatial Analysis and Indoor Radon Map of the Area

**Figure 11:**
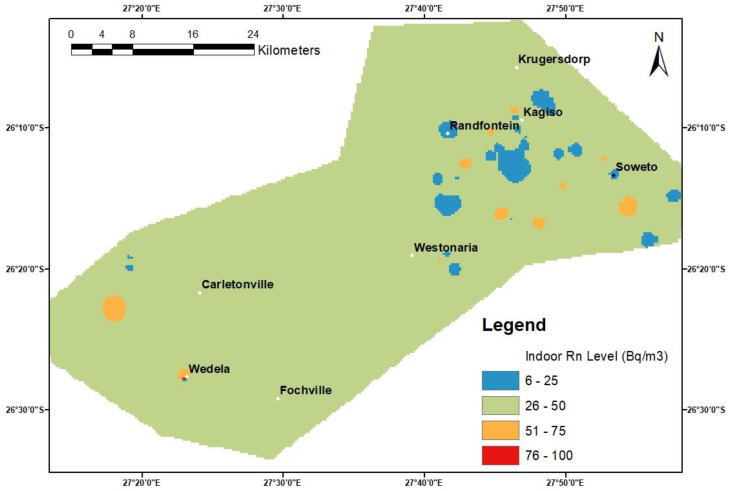
Indoor radon spatial map of the study area.

**Figure 12:**
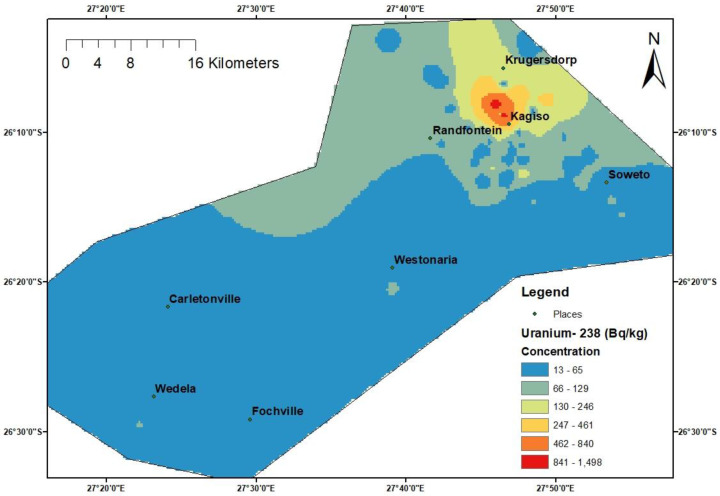
Uranium-238 spatial map of the study area.

This study established a radon map for the study area using the average indoor radon concentrations that were acquired to observe the spatial distribution of radon concentrations in dwellings across various areas. The findings indicate that, on average, most of the dwellings in the area are predicted to have indoor radon concentrations that fall in the range of 26 to 50 Bq/m^3^ (Figure 11). It is observed that more diverse clusters of variable indoor radon concentrations are found in the upper eastern residential areas of the study area. These clusters and the general spatial trend observed, to a greater extent, correlate with the surface uranium-238 concentrations found in the study area (Figure 12). This provides an indication that the concentrations of the parent radionuclides in the adjacent rocks and soils have a positive influence on indoor radon levels. Overall, the range of indoor radon concentrations from the radon map of this study, which was constructed using the direct indoor radon data, agrees with the radon potential map predicted using the estimated uranium concentration in various rocks, which predicts indoor radon levels between 0 and 100 Bq/m^3^ in most parts of the study area [51].

## 4. Discussion

Based on the previous study that measured radon concentrations in outdoor environments in some parts of the West Rand District, it was found that the highest concentrations were associated with mine tailings dams, which also contributed towards increasing radon levels in areas within proximity [26]. In the case of the indoor radon concentrations measured in this study, the levels were generally low and below the WHO reference value of 100 Bq/m^3^, except in one dwelling. This trend was observed even in dwellings which are in the closest proximity to tailings with high radon exhalations and high concentrations of radon parent radionuclides. This could indicate that the enclosed nature of building structures serves as a shielding mechanism to control the entry of radon into the dwellings and limit air circulation between outdoor and indoor air to allow for an asymptotic balance. This shows that, to a greater extent, the building characteristics of dwellings determine the entry of radon into buildings from outdoor air. In addition, the fact that some of the mine tailings are covered with vegetation may minimize the dispersion of radon gas from the tailings sources. In general, the results of the long-term measurements of this study ascertain that the mine tailings distributed across the West Rand District and Soweto do not result in substantial radon levels in dwellings. The only exception where the impact of the mine tailings was directly observed was in a dwelling in Kagiso (Krugersdorp) that is built on soil mixed with eroded materials from the tailings, which recorded the highest-activity concentration of 124 Bq/m^3^. This, therefore, provides insights that mine tailings may impact indoor radon concentrations in cases where dwellings are built directly on mining residual materials or on soil that is contaminated by tailings, as this will allow for the direct ingress of the gas into the dwellings. On the basis of the results presented in this study, it could be deduced that the types of bedrocks found in the area, which then primarily determine the concentration of the radon parent radionuclides found on the surface, the changes in meteorological conditions, the characteristics of the building structures and the rate of ventilation in a household, have a synergistic effect on indoor radon levels.

### 4.1. Comparison of the Findings of This Study with Previous Indoor Radon Studies

Most of the published studies in South Africa conducted indoor radon measurements over a short period [52,53,54,55,56]. There are only a few studies which performed long-term measurements of indoor radon levels [57,58]. When the indoor radon activity concentrations obtained in this study are compared with that of the large-scale study of Leuschner et al. [57], it is observed that the radon concentrations found in the dwellings monitored in this study are lower (Table 1). The differences may be due to the spatial variability of radon parent radionuclides found in the surveyed areas, as the measurements were taken in various locations with variable geological formations. In Soweto, where the sub-areas surveyed are similar to those covered in this study, it was found that the average activity concentration obtained during winter was 56 Bq/m^3^ [57], and in this study, the summer measurements averaged at 34 Bq/m^3^ in Soweto (Table 1). This results in a seasonal variation ratio of 1.64, which is very close to the average winter-to-summer ratio of 1.7 determined in this study. This therefore ascertains that, on average, the indoor radon levels during winter in the studied area are almost twice the summer activity concentrations.

### 4.2. Indoor Radon on Public Health

Radon is a health concern to the public because when it is inhaled, the ionizing alpha particles emitted by its short-lived decay products, ^218^Po and ^214^Po, interact with tissues in the lungs and lead to DNA damage. A single alpha particle has the potential to result in major genetic damage to a cell, therefore making it possible for radon-related DNA damage to happen at any level of exposure. There is no known threshold concentration below which exposure to radon does not present a potential risk for developing lung cancer [7]. The findings of this study showed that the exposure levels are below the proposed WHO reference level of 100 Bq/m^3,^ except at one location. From a public health perspective, a reference level of 100 Bq/m^3^ is justified because an effective reduction in radon-related health hazards for a population is expected [7]. This therefore means that the level of risk from indoor radon exposure in the studied areas is low, based on the overall activity concentrations found. However, it should be noted that radon is considered as a carcinogen at all exposure levels [10], and there is still a potential risk of lung cancer development. This is because the development of lung cancer from radon exposure is associated with multiple attributes, such as the duration of exposure, smoking habits and exposure to other carcinogens [6,8]. Moreover, the characteristics of the lung of an individual determine the amount of dose received from radon exposure [8]. Studies have revealed that radon-induced lung cancers are statistically higher in smokers than non-smokers [9,10,59]. This is because the particulates from smoking enhance the deposition of more radon progenies, thus increasing the radiation dose received by the lung cells [8]. In the dwelling where a value of 124 Bq/m^3^ was obtained, the ventilation of the house should be increased to reduce the indoor radon levels. It is important that members of the public keep the radon levels in their dwellings low as reasonably achievable and minimise their exposure to other lung cancer-causing agents.

## 5. Conclusions

In this study, a regional mapping survey was conducted to assess indoor radon exposure levels in a historically mined area dominated by gold and uranium tailings dams. Based on the findings of this study, there are no indications of elevated indoor radon concentrations that require regulatory control as of now. Although the indoor radon concentrations found in this study are low, it should be noted that long-term exposure to low concentrations may lead to increased lung cancer risks. The findings confirm that the mine tailings dams distributed in the study area do not have a significant impact on indoor radon concentrations, except for when houses are built directly on contaminated sites. It is therefore recommended that future studies should conduct extensive indoor radon measurements in residential homes that have been constructed directly on legacy sites related to previous gold and uranium mining activities.

It was found that the main contributing source of indoor radon is the geological formations and soil beneath the dwellings. This study further demonstrated that, apart from the primary sources of indoor radon, additional factors such as seasonal variations, ventilation rate and the age of the buildings have a net effect on the amount of radon that accumulates in indoor environments. The indoor radon spatial map produced in this study indicates that the area is mostly dominated by low-radon zones with activity concentrations of less than 100 Bq/m^3^. This may be attributable to good ventilation conditions prevailing in the dwellings where measurements were taken. It is recommended that the effect of ventilation on indoor radon should be quantified in future studies to better understand its impact on radon concentrations. 

The findings of this study will bring awareness to the communities residing in the studied areas and to the public at large on indoor radon exposure levels in gold mining areas and the health risks associated with radon exposure. The findings will further inform the National Nuclear Regulator (NNR) decision-making process on the control and regulation of radon exposures in residential homes in and around NORM contaminated sites in South Africa. Overall, the data acquired in this study will contribute to the NNR national database of indoor radon levels in South Africa and serve as a baseline reference to evaluate the impact of gold mine tailings on indoor radon concentrations in Gauteng Province. 

## Figures and Tables

**Table 1:** Statistical summary of indoor radon concentrations (Bq/m^3^).

Summer Indoor Radon Measurements							
Region	Area	Statistical Summary of Radon Concentrations (Bq/m^3^)					
		No. of Measurements (No. of Dwellings)	Min	Median	Mean	Max	SD
West Rand District	Krugersdorp	59 (33)	<LLD	21	24	67	14
	Randfontein	15 (9)	5	23	22	61	16
	Westonaria	13 (7)	15	27	28	43	10
	Carletonville	25 (13)	5	37	36	69	17
Johannesburg Metropolitan	Soweto	41 (23)	15	29	34	71	17
	All Areas	153 (85)	<LLD	25	29	71	16
**Winter Indoor Radon Measurements**							
**Region**	**Area**	**Statistical Summary of Radon Concentrations (Bq/m^3^)**					
		**No. of measurements (No. of dwellings)**	**Min**	**Median**	**Mean**	**Max**	**SD**
West Rand and Soweto	All areas	65 (35)	11	41	46	124	21

LLD: Lower limit of detection.

**Table 2:** Comparison of winter and summer indoor radon concentrations for two rooms in the same dwelling.

Season	No of Measurements	Ratio of Indoor Radon Concentration for Two Rooms in the Same Dwelling		
		Mean	Median	Range
Summer	61	2.8	2.1	1.0–12.8
Winter	30	1.2	1.2	1.0–2.0

**Table 3:** Correlation of indoor radon with distance from the mine tailings.

Distance of Measured Dwellings from the Mine Tailings Zones (km)	Activity Concentration of Indoor Radon in Measured Dwellings (Bq/m^3^)					
	No of Measurements	Min	Median	Mean	Max	SD
Dwellings: <0.5 km	19	9	22	33	124	27
Dwellings: 0.5 km–2 km	31	5	31	34	80	18
Dwellings: 2 km–3.5 km	27	11	32	35	71	16
Dwellings: 3.5 km–5 km	20	6	35	37	82	18
Dwellings: 5 km–6.5 km	13	5	25	32	69	18
Dwellings: 6.5 km–8 km	5	21	43	37	43	10
Dwellings: >8 km	3	29	34	34	41	6

## Data Availability

Data are contained within the article.
